# High-Throughput Assay of Levansucrase Variants in Search of Feasible Catalysts for the Synthesis of Fructooligosaccharides and Levan

**DOI:** 10.3390/molecules19068434

**Published:** 2014-06-20

**Authors:** Karin Mardo, Triinu Visnapuu, Maria Gromkova, Anneli Aasamets, Katrin Viigand, Heiki Vija, Tiina Alamäe

**Affiliations:** 1 Department of Genetics, Institute of Molecular and Cell Biology, University of Tartu, Riia 23, 51010 Tartu, Estonia; E-Mails: kmardo@ut.ee (K.M); triinu.visnapuu@ut.ee (T.V.); manjunja@ut.ee (M.G.); anneli.aasamets@gmail.com (A.A); katrin66@ut.ee (K.V.); 2 National Institute of Chemical Physics and Biophysics, Akadeemia tee 23, 12618 Tallinn, Estonia; E-Mail: heiki.vija@kbfi.ee

**Keywords:** levansucrase, fructooligosaccharides, levan, *Pseudomonas syringae*, cell permeabilization, Thermofluor, prebiotics

## Abstract

Bacterial levansucrases polymerize fructose residues of sucrose to β-2,6 linked fructans—fructooligosaccharides (FOS) and levan. While β-2,1-linked FOS are widely recognized as prebiotics, the health-related effects of β-2,6 linked FOS are scarcely studied as they are not commercially available. Levansucrase Lsc3 (Lsc-3) of *Pseudomonas syringae* pv. tomato has very high catalytic activity and stability making it a promising biotechnological catalyst for FOS and levan synthesis. In this study we evaluate feasibility of several high-throughput methods for screening and preliminary characterization of levansucrases using 36 Lsc3 mutants as a test panel. Heterologously expressed and purified His-tagged levansucrase variants were studied for: (1) sucrose-splitting activity; (2) FOS production; (3) ability and kinetics of levan synthesis; (4) thermostability in a Thermofluor assay. Importantly, we show that sucrose-splitting activity as well as the ability to produce FOS can both be evaluated using permeabilized levansucrase-expressing *E. coli* transformants as catalysts. For the first time we demonstrate the key importance of Trp109, His113, Glu146 and Glu236 for the catalysis of Lsc3. Cost-effective and high-throughput methods presented here are applicable not only in the levansucrase assay, but have a potential to be adapted for high-throughput (automated) study of other enzymes.

## 1. Introduction

Levansucrases (EC 2.4.1.10) are bacterial extracellular enzymes that convert sucrose into β-2,6- linked fructooligosaccharides (FOS) of varied chain length and high-molecular weight levan [1]. These enzymes are present in many plant-related bacteria such as *Pseudomonas syringae* [2,3,4], *Gluconacetobacter diazotrophicus* [5], *Zymomonas mobilis* [6] and *Erwinia amylovora* [7,8,9], but also in *Bacillus subtilis*, *B. megaterium* [10,11] and several lactic acid bacteria such as *Lactobacillus sanfranciscensis*, *L. reuteri* and *Leuconostoc mesenteroides* [12,13,14]. 

FOS which are derived from plant storage polysaccharide inulin (a β-2,1 linked fructan) are already widely recognized as prebiotics [15,16]. They are industrially produced from plant sources and used in various food- and health-related products. On the contrary, other types of FOS are not commercially available and therefore their biological effects are scarcely studied. Still, a few papers, for example [17], report that β-2,6-linked (levan-type) FOS are selectively fermented by bifidobacteria showing even stronger prebiotic effects than their β-2,1 linked counterparts. Neokestose, a fructosylglucosylfructoside produced from sucrose by a fungus *Xanthophyllomyces dendrorhous*, also showed a bifidogenic effect on human gut microbiota [18]. Notably, a recent paper by Marsh and coworkers states that water kefir grains originating from different regions of the world contain *Z. mobilis* as main bacterial component [19]. As *Z. mobilis* possesses a levansucrase, water kefir, a popular healthy drink produced by fermentation of sucrose-containing water with water kefir grains as a starter, most likely contains levan and FOS. The prebiotic effect of polymeric fructans (inulin and levan) on lactobacilli and bifidobacteria is most probably assisted by other bacteria in the gut that degrade these large molecules to oligomers. For further study of the physiological effects of β-2,6-linked FOS and levan, biotechnologically feasible production systems applying wild-type enzymes or selected mutant variants should be established. 

We have cloned and heterologously expressed three genomic levansucrase genes *lsc1*, *lsc2* and *lsc3* (also designated as *lsc-1*, *lsc-2* and *lsc-3*) from a plant pathogen, *Pseudomonas syringae* pv. tomato [4]. The respective proteins have highly similar sequences and general catalytic properties [4,20]. We have shown that purified Lsc3 protein has a very high catalytic constant (k_cat_ 504.4 1/s) [21]. A higher k_cat_ (2272 1/s) has been recorded only for the levansucrase of *B. megaterium* [11] whereas levansucrases of *G. diazotrophicus* and *Z. mobilis* have eight and 18 times lower k_cat_ values than Lsc3, respectively [5,6]. Lsc3 is a very efficient polymerizer, producing two types of fructans from sucrose: high-molecular weight levan and short-chain FOS [21,22]. The spectrum of FOS produced by Lsc3 is highly similar to that of a prebiotic inulin-type FOS mixture (P95 from Orafti, Beneo, Belgium) as verified using different analysis methods: thin layer chromatography (TLC), nanoelectrospray ionization mass spectrometry (nanoESI MS) [22] and high-performance liquid chromatography (HPLC) [23]. Importantly, Lsc3 transfructosylated eleven out of twelve nonconventional acceptor substrates tested by us. Among them, sorbitol, xylobiose, galacturonic acid, mannitol, xylitol and methyl-glucopyranoside were shown to serve as fructosyl acceptors for levansucrases for the first time [21].

In the search, isolation and characterization of levansucrase mutants, we have elaborated and applied several high-throughput methods. Firstly, for the selection of random mutants of the Lsc3 protein, we introduced a microplate-based assay of levansucrase activity on permeabilized cells of levansucrase-expressing *E. coli* as a catalyst. This method was further applied for preliminary study of site-directedly mutated Lsc3 variants [21]. As the majority (88%) of levansucrase activity was detected in the cytoplasmic fraction of levansucrase-expressing *E. coli* and only 12% in the periplasm [4], permeabilization is needed to disclose also the activity of the cytoplasmic fraction of the protein [20]. Secondly, we introduced a microplate-based assay of levan production kinetics to characterize polymerization ability of the Lsc3 mutants [21]. Thirdly, for the first time we applied NanoMate robot-assisted electrospray ionization coupled with high-capacity ion trap mass spectrometry for the analysis of Lsc3-produced homo- and heterooligofructans in underivatized form [21,22].

In the current work we introduced a set of high-throughput and cost-saving methods feasible for levansucrase assay. These methods were evaluated on a panel of random and site-specifically constructed Lsc3 mutants. Several of these mutants have been described earlier, but novel variants were also included. The wild-type Lsc3 was be used as a reference. We assayed following biochemical properties of the purified levansucrases: (1) sucrose-splitting activity, also referred to as total activity; (2) the amount and spectrum of FOS produced; (3) ability and kinetics of levan synthesis; (4) thermal stability of levansucrases. In addition, we evaluated some of these characteristics using permeabilized cells of levansucrase-expressing *E. coli* as catalysts. The results retrieved from high-throughput assays were compared with those obtained by using conventional more laborious methods.

## 2. Results and Discussion

### 2.1. The Lsc3 Mutants Used in This Study

**Figure 1:**
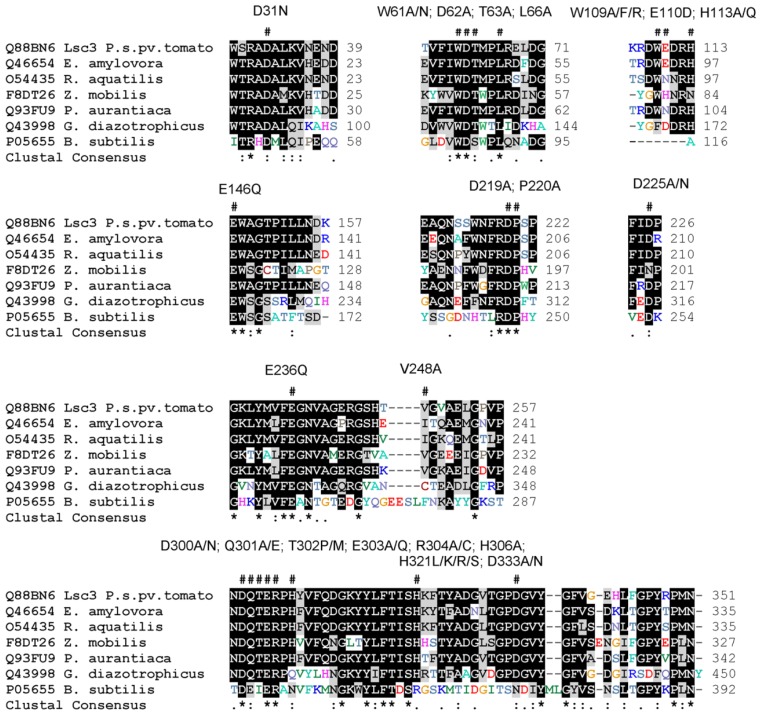
Lsc3 mutants with substituted positions designated as # above the MUSCLE [26] alignment of levansucrases from *Pseudomonas syringae* pv. tomato (Lsc3), *Erwinia amylovora*, *Rahnella aquatilis*, *Zymomonas mobilis*, *Pseudomonas chrororaphis* subsp. *aurantiaca, Gluconacetobacter diazotrophicus* and *Bacillus subtilis*.

Lsc3 variants addressed in the study include the wild-type Lsc3 and its thirty six mutants. Twenty two of them were previously characterized by us. Among those are the inactive mutants of the catalytic triad Asp62Ala, Asp219Ala and Glu303Ala [23], mutants with significantly decreased polymerizing ability (Thr302Pro, Gln301Ala and substitution mutants of His321 with Arg, Leu, Lys or Ser) [21,23], mutants Trp61Ala, Trp61Asn, Arg304Cys and Arg304Ala which are strongly hampered in sucrose-splitting as well as polymerization abilities [23] and some others exhibiting moderate changes compared to the wild-type Lsc3. Fourteen mutants of Lsc3 are described here for the first time. Among those are so-called “Yanase mutants”. Inspired by a superior paper by Yanase *et al.* [6] on mutational analysis of the *Zymomonas mobilis* levansucrase (LevU), we constructed homologues of Trp80Arg, Glu117Gln, Glu211Gln, Val223Ala and Asp308Asn mutants of *Z. mobilis* enzyme. Corresponding mutants of Lsc3 are Trp109Arg, Glu146Gln, Glu236Gln, Val248Ala and Asp333Asn. To gain more information on the functions of these positions, some additional substitutions were made. Therefore, Trp109 in Lsc3 was also replaced with Ala and Phe, and Asp333 with Ala. Several novel mutants of Lsc3 studied here originate from random mutagenesis, the method of which was described by us earlier [21]. All Lsc3 variants addressed in this work and respective mutagenic oligonucleotides are listed in Supplementary Table S1. Mutant variants are also designated in Figure 1 above the alignment of the levansucrases. The mutant *lsc3* genes were cloned into pURI3 vector for overexpression in *E. coli* as N-terminally His_6_-tagged fusion proteins ([24,25]; see Section 3.1 and Section 3.2).

**Figure 2:**
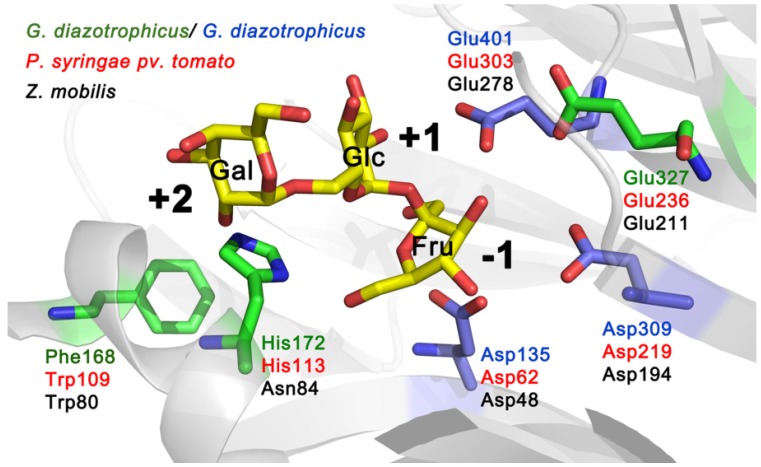
Catalytic centre of *G. diazotrophicus* levansucrase LsdA (PDB ID: 1W18; [5]) with side chains of catalytic triad residues (Asp135, Asp309 and Glu401) indicated in blue. The structure of the protein was visualized using PyMol [27]. Raffinose (consisting of galactose, glucose and fructose residues) shown in substrate-binding pocket originates from the structure of the *B. subtilis* levansucrase SacB in complex with raffinose (PDB ID: 3BYN) that was superimposed with the LsdA structure. Positions of LsdA equivalent to Trp109, His113 and Glu236 of Lsc3 of *P. syringae* pv. tomato are shown on the model. Respective amino acids of levansucrases of different bacteria are shown in different colour: LevU of *Z. mobilis* (**black**), Lsc3 of *P. syringae* pv. tomato (**red**) and LsdA of *G. diazotrophicus* (**green** and **blue**).

Figure 2 shows the location of amino acids equivalent to Trp109, His113 and Glu236 of Lsc3 on a 3D model of *G. diazotrophicus* enzyme LsdA. Trp109 and Glu236 correspond to Trp80 and Glu211 in *Z. mobilis* levansucrase (Figure 1). The Glu211Gln mutant showed vastly reduced polymerizing ability and Trp80Arg mutant was unable to synthesize polymeric levan [6]. As we show in this study, mutation of His113 in Lsc3 (we studied His113Gln and His113Ala substitutions) has a strong negative effect on the catalysis (Section 2.3; for further discussion see Section 2.7). The importance of the above-mentioned positions can be predicted from the location of respective residues in the substrate-binding pocket (Figure 2).

### 2.2. The Simplest Way to Detect Levansucrase Activity—Assay of the Growth Phenotype of Levansucrase-Expressing Bacteria on Agar Plate Containing Sucrose

**Figure 3:**
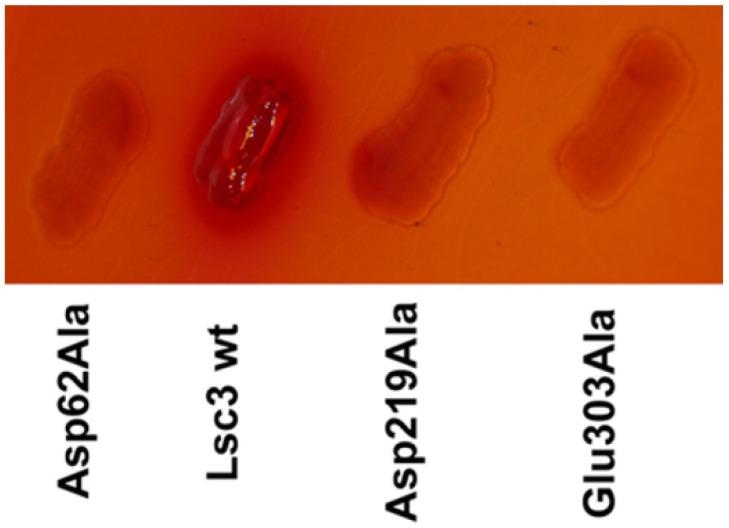
Levan synthesis phenotype of *E. coli* BL21(DE3) expressing wild-type Lsc3 and alanine replacement mutants of the catalytic triad amino acids. Transformants were streaked onto MacConkey + Amp medium containing 10% sucrose and 1 mM IPTG to induce the T7 polymerase and grown at 37 °C (see Section 3.2).

Levansucrase-possessing bacteria have mucoid colonies when grown on sucrose-containing agar plate due to the synthesis of levan. This feature can be used to detect and identify bacteria that produce a levansucrase, but also to select levansucrase mutants through heterologous expression in *E. coli* [6,20,28]. We have used this simple and informative method in the cloning of *lsc1*, *lsc2* and *lsc3* genes of *P. syringae* pv. tomato [4] and selection of random mutants of the Lsc3 protein [21]. Figure 3 shows growth phenotype of *E. coli* transformants expressing wild-type Lsc3 and its catalytic triad mutants growing on MacConkey medium containing 10% sucrose. Only wild-type Lsc3 produces levan giving a mucoid phenotype to the streaks of respective transformant. Also, only in case of wild-type Lsc3 expression, acidification of the medium (pink colour around the streak) due to sugar fermentation is visible.

### 2.3. Total Levansucrase Activity Can Be Reliably Measured on Microplates not only Using Purified Proteins, but also Implementing Permeabilized E. coli Transformants as Catalysts

**Figure 4:**
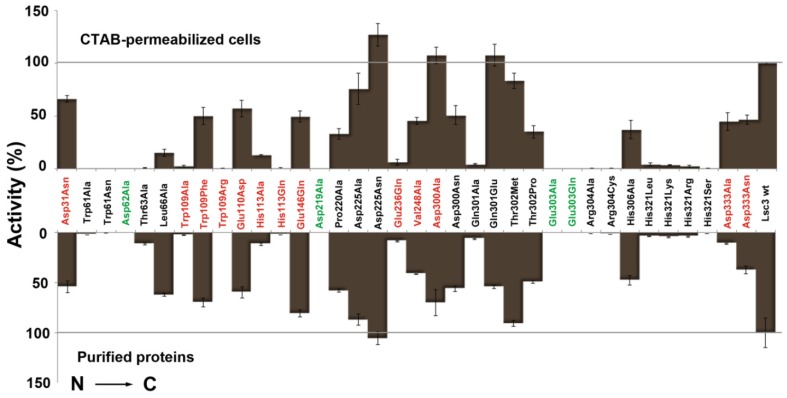
Quantitative evaluation of the sucrose-splitting (total) levansucrase activity on microplates. Either CTAB-permeabilized *E. coli* transformants expressing a certain Lsc3 variant (upper panel) or respective purified proteins (lower panel) were used as catalysts. Respective methods are described in Section 3.3 and Section 3.4. Mutants of catalytic triad positions are designated in green and Lsc3 mutants studied for the first time in red. Mutant Glu303Gln falls into both categories. The activities of mutant enzymes are expressed as percentage of respective value of wild-type Lsc3 taken as 100% (grey lines). Total activity of purified wild-type Lsc3 protein was 461.6 ± 95.5 U/mg. In the assay on permeabilized cells, the absorption values at 500 nm indicating glucose concentration were normalized to cell suspension densities and the value obtained for wild-type Lsc3 was taken as 100%. At least three parallel measurements for each levansucrase variant were made and mean values with respective standard deviations are shown.

Most often, enzyme activities are measured by performing reactions in a volume of 1–2 mL. This approach needs a high amount of protein and chemicals and is usually quite time-consuming and costly. Microplate-aided assays save time, money and can be robotized. Total activity of levansucrases is traditionally evaluated according to the velocity of sucrose-splitting reaction. This reaction is required for both sucrose hydrolysis as well as transfructosylation reaction. Each act of sucrose splitting releases a glucose molecule that can be quantified in several ways. We have been using the Glucose liquicolor assay. As described by the manufacturer (Human GmbH, Wiesbaden, Germany), this procedure relies on oxidation of glucose by glucose oxidase, yielding hydrogen peroxide which reacts under catalysis of peroxidase with phenol and 4-aminophenazone to yield a reddish quinoneimine product, concentration of which is measured at 500 nm. We routinely determine kinetic constants of sucrose-splitting reaction of levansucrases by performing reaction in Eppendorf tubes with 1 mL volume of reaction mixture. At fixed time points, small samples are withdrawn for the estimation of liberated glucose. We have earlier used this approach to determine the K_m_ and V_max_ values of sucrose-splitting reaction of levansucrases [4,21,23] and applied the same method here (see Section 3.8). Using a set of 36 Lsc3 mutants with largely varied catalytic activity, we show here that sucrose-splitting activity of the levansucrases can be reliably measured in a high-throughput way, *i.e.*, on microplates. Moreover, we demonstrate that sucrose splitting by a levansucrase can also be evaluated on the permeabilized *E. coli* transformants expressing respective protein. Figure 4 compares the results obtained by these two methods. One can see that they correlate fairly well. Our levansucrase assay on permeabilized *E. coli* transformants presumes similar expression level for different Lsc3 variants in the host. Therefore, all induced *E. coli* cultures subjected to levansucrase activity assay were studied for levansucrase expression and similar expression levels for different levansucrase variants were detected. From every induced culture, equal amounts were analysed by SDS-PAGE and the intensity of the bands corresponding to levansucrase protein was compared after staining of the gels with Coomassie Brilliant Blue (data not shown).

To permeabilize the *E. coli* cells, we used 0.1% cetyltrimethylammonium bromide (CTAB) (Section 3.3) that has only minimal activity-reducing effect on levansucrases. Notably, we have earlier used this surfactant not only for levansucrase activity assay on recombinant *E. coli* [23], but also to evaluate activities of maltase, β-glucuronidase and enzymes of methanol oxidation in yeast cell suspensions [29,30]. Earlier, de Abreu * et al.* [31] described a high-throughput microplate assay of *Schwanniomyces occidentalis* β-fructofuranosidase mutants. Gene library of respective genes was expressed in an invertase-negative mutant of *Saccharomyces cerevisiae* to reveal the protein variants with enhanced transfructosylation ability [31]. In that case, permeabilization of the yeast cells was not required as invertase is a cell surface-bound protein.

**Figure 5:**
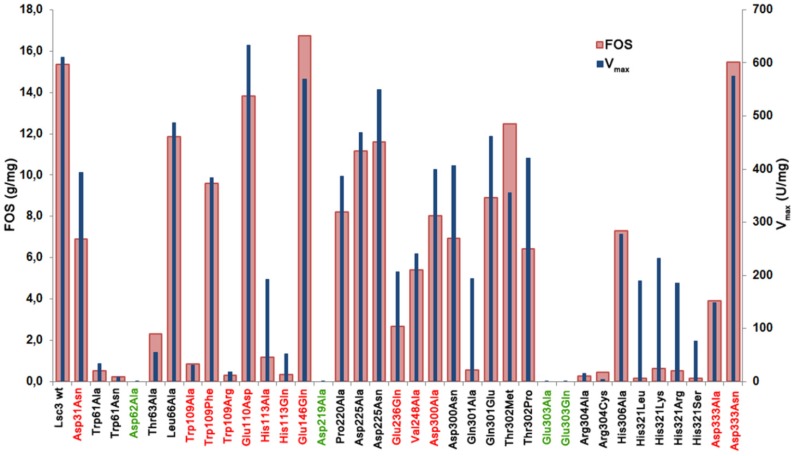
The amount of total FOS (g/mg of protein) produced from 1.2 M sucrose in a 20 h reaction by purified wild-type Lsc3 and thirty six mutant proteins. Mutants of catalytic triad positions are shown in green and the mutants studied in this work for the first time are in red. V_max_ (U/mg) of sucrose-splitting reaction is plotted for comparison. Mean values of at least two independent experiments are shown. Standard deviation was less than 15%.

Figure 4 indicates that even a simple levansucrase activity assay on permeabilized *E. coli* clearly discloses catalytically disabled mutants Trp61Ala, Trp61Asn, Asp62Ala, Asp219Ala, Glu303Ala, Glu303Gln, Arg304Ala, Arg304Cys, His321Arg, His321Leu, His321Lys and His321Ser [21,23]. Concerning novel mutants, Trp109Ala, Trp109Arg, His113Gln and Glu236Gln stick out as the variants with significantly reduced sucrose-splitting ability. For comparison, V_max_ values of the sucrose-splitting reaction determined with purified proteins are presented in Figure 5. Some Lsc3 mutants, for example Glu236Gln and substitution mutants of His321, have highly reduced affinity for sucrose (Table 1). In consequence of that, the sucrose-splitting ability of these mutants is somewhat underestimated in microplate assay which was conducted with 100 mM sucrose (Figure 4, Table 1).

**Table 1:** Affinity (K_m_) for sucrose and polymerizing properties of Lsc3 and its mutants. K_m_ and TA were determined as described in Section 3.8 and the reaction products were analysed by HPLC (Section 3.9). The degree of polymerization (DP) of detected FOS is also presented. Reactions were performed either with purified proteins (see Section 3.6) or permeabilized cells of levansucrase-expressing *E. coli* (see Section 3.5). In the TA assay, 100% corresponds to the hypothetic situation where no free fructose is produced as a reaction product and all fructose residues of reacted sucrose molecules are incorporated into polymerization products FOS and levan. The mean values and standard deviation values of at least two independent experiments are shown.

Levansucrase	K_m_ (mM)	Transfructosylation Activity (TA; %) Permeabilized Cells/Purified Protein	Degree of Polymerization (DP) Permeabilized Cells/Purified Protein
Lsc3 wt	18.5 ± 2.5	71 ± 1/74 ± 1 ^a^	3–7/3–7 ^a^
Asp31Asn	14.9 ± 4.6	65 ± 4/70 ± 3	3–6/3–6
Trp61Ala	143.4 ± 6.1	ND/69 ± 1 ^a^	ND/3–6 ^a^
Trp61Asn	869.3 ± 104.0	ND/51 ± 1 ^a^	ND/3–4 ^a^
Asp62Ala	20.7 ± 2.1	ND/ND	ND/ND
Thr63Ala	15.9 ± 1.8	75 ± 3/71 ± 3 ^a^	3–6/3–7 ^a^
Leu66Ala	27.3 ± 4.2	69 ± 4/73 ± 2 ^a^	3–6/3–7 ^a^
Trp109Ala	29.8 ± 3.3	72 ± 6/77 ± 1	3–5/3–7
Trp109Phe	9.6 ± 0.6	62 ± 3/74 ± 1	3–7/3–7
Trp109Arg	249.1 ± 38.5	ND/40 ± 1	ND/3
Glu110Asp	57.6 ± 9.7	57 ± 4/70 ± 1	3–7/3–7
His113Ala	170.4 ± 17.0	43 ± 1/41 ± 6	3–5/3–6
His113Gln	190.1 ± 28.3	65 ± 5/51 ± 3	3–5/3–6
Glu146Gln	40.6 ± 5.0	63 ± 5/76 ± 1	3–6/3–6
Asp219Ala	43.4 ± 10.2	ND/ND	ND/ND
Pro220Ala	23.9 ± 2.3	71 ± 2/75 ± 2 ^a^	3–6/3–6 ^a^
Asp225Ala	13.7 ± 1.2	61 ± 1/71 ± 2 ^a^	3–6/3–7 ^a^
Asp225Asn	18.8 ± 0.8	57 ± 2/71 ± 2 ^a^	3–6/3–7 ^a^
Glu236Gln	267.1 ± 40.2	46 ± 1/50 ± 4	3–5/3–6
Val248Ala	14.1 ± 1.1	63 ± 2/72 ± 1	3–7/3–6
Asp300Ala	19.4 ± 1.3	53 ± 4/58 ± 1	3–7/3–8
Asp300Asn	50.7 ± 5.4	52 ± 2/60 ± 1 ^a^	3–9/3–10 ^a^
Gln301Ala	313.7 ± 30.0	24 ± 5/24 ± 1 ^a^	3–4/3–4 ^a^
Gln301Glu	23.6 ± 1.7	34 ± 8/45 ± 1 ^a^	3–5/3–5 ^a^
Thr302Met	15.1 ± 1.8	56 ± 1/70 ± 1 ^a^	3–6/3–6 ^a^
Thr302Pro	42.5 ± 6.7	39 ± 5/52 ± 5 ^a^	3–6/3–6 ^a^
Glu303Ala	27.1 ± 5.5	ND/ND	ND/ND
Glu303Gln	129.6 ± 9.6	ND/ND	ND/ND
Arg304Ala	66.0 ± 9.8	ND/70 ± 2 ^a^	ND/3–6 ^a^
Arg304Cys	12.5 ± 1.1	ND/69 ± 1 ^a^	ND/3–4 ^a^
His306Ala	21.2 ± 2.4	70 ± 5/72 ± 1 ^a^	3–7/3–7 ^a^
His321Leu	352.1 ± 41.7	17 ± 1/20 ± 5	3/3
His321Lys	529.5 ± 68.3	30 ± 1/27 ± 2	3–4/3–4
His321Arg	451.0 ± 36.8	36 ± 1/25 ± 1	3/3–4
His321Ser	503.3 ± 87.8	31 ± 4/23 ± 3	3–4/3–4
Asp333Ala	27.0 ± 2.9	65 ± 3/68 ± 1	3–5/3–5
Asp333Asn	41.3 ± 4.2	67 ± 3/80 ± 1	3–7/3–7

^a^ data from Ref. [23]; ND—not detected; value under detection limit.

### 2.4. The Ability of FOS Production by Levansucrases Can be Evaluated on Microplates

Production of β-2,6 linked FOS is important because of their potential biological (prebiotic) effects. They are not produced commercially and are therefore practically impossible to purchase. The β-2,6 linked FOS have been produced for prebiotic efficiency studies in small amounts by controlled chemical hydrolysis of bacterial levan and isolation of the oligosaccharide fractions [17]. However, some levansucrases, for example LsdA of *G. diazotrophicus*, produce mostly short-chain products from sucrose and only a low amount of levan [32,33,34]. Levansucrases with this ability are potential catalysts for large-scale synthesis of β-2,6 linked FOS. We showed that Lsc3 protein of *P. syringae* pv. tomato is capable of FOS synthesis from sucrose, raffinose and sugar beet molasses. A high substrate concentration (600 mM and higher) and prolonged reaction time are required for FOS synthesis [22,23]. In the search of FOS-producing levansucrases, high-throughput methods for the analysis are preferred. We show here that the ability of levansucrases to produce FOS can be evaluated on microplates. Moreover, even permeabilized *E. coli* culture expressing a levansucrase can be reliably applied for FOS production assay (see Supplementary Figure S1). Indeed, if a levansucrase-expressing *E. coli* was permeabilized by 0.1% CTAB and incubated on a microplate in buffer with 1 M of sucrose for 20 h, FOS were produced. The FOS spectrum characteristic for Lsc3 variants of our panel was determined by HPLC (see Section 3.9) and results are presented in Table 1. Respective spectra from reactions with purified proteins are given for comparison. Table 1 also shows transfructosylating activity (TA) of the levansucrases.

The liberation of glucose from a sucrose-splitting reaction shows the total levansucrase activity because glucose molecules are by-products of the reaction and are not polymerized. Fructosyl residues can be transferred to water, resulting in free fructose (hydrolytic activity) or to acceptor molecules other than water (sucrose and fructans), resulting in polymerization products such as FOS and levan (transfructosylating activity). The amount of free glucose and fructose in the reaction mixture is measured and TA is calculated according to the formula: ([Glc] − [Fru_F_])/[Glc]) × 100, where [Glc] indicates the content of free glucose and [Fru_F_] the content of free fructose in the reaction mixture. Therefore TA indicates the percentage of fructosyl residues that levansucrase protein is using for transfructosylation [21]. Low TA value means that most of the fructosyl residues from splitted sucrose are transferred to water.

Table 1 shows that the size range of FOS produced can be detected by both high-throughput methods. For example, the Asp300Asn mutant stands out in both assays as the producer of FOS with extended chain length, up to degree of polymerization (DP) 9–10. The mutants synthesizing only short-chain FOS, with DP up to 4 (e.g., Gln301Ala and substitution mutants of His321) were also disclosed in both assays. In several cases, FOS production could not be detected in the assay on permeabilized transformants. In case of mutants with largely decreased catalytic activity, such as Trp61Ala, Trp61Asn, Trp109Arg, Arg304Ala and Arg304Cys (see activity data on Figure 4 and V_max_ data in Figure 5), FOS were detected only in reactions that were performed with an increased amount (100 µg/mL) of purified protein. Figure 5 shows data on total FOS production by Lsc3 variants per 1 mg of pure protein.

V_max_ values of sucrose splitting for respective mutants are also included. Comparison of these two characteristics indicates that in case of mutants Asp31Asn, Glu110Asp, His113Ala, Glu236Gln, Asp300Ala, Asp300Asn, Gln301Ala, Thr302Pro and substitution mutants of His321 (His321Arg, His321Leu, His321Lys, His321Ser), FOS production is substantially more affected that the ability to split sucrose, meaning that these positions are specifically important for polymerization reaction. The mutants Glu146Gln, Thr302Met and Asp333Asn with fairly good sucrose-splitting activity and slightly enhanced FOS production (Figure 5) can be considered as promising candidates for enzymatic synthesis of FOS for biotechnological applications.

### 2.5. Online Assay of Levan Synthesis Kinetics on a Microplate

**Figure 6:**
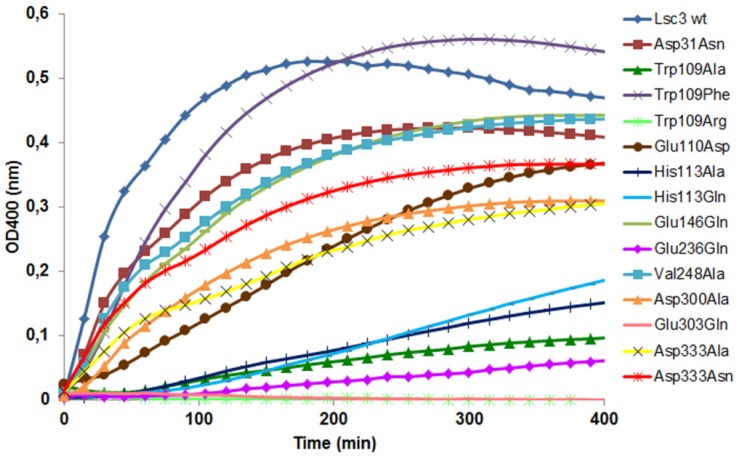
Time course of levan synthesis by the wild-type Lsc3 protein (Lsc3 wt) and fourteen mutants. Reaction was conducted at 37 °C on flat-bottom polystyrole microplates in total volume of 200 μL per well. Reaction mixture contained 600 mM of sucrose in McIllvaine’s buffer (pH 6.0) and 10 μg/mL of purified protein. Increase of turbidity was measured at 400 nm every 15 min using Infinite M200 PRO microplate reader. Mean values of four parallel measurements are shown. Standard deviation was less than 10%.

In this high-throughput procedure, the increase of turbidity due to levan formation is recorded online [21]. Here, we will present data on levan synthesis kinetics of fourteen novel Lsc3 mutants. Figure 6 shows that two mutants, Trp109Arg and Glu303Gln, do not produce levan. Glu303 is acid-base catalyst of Lsc3 [23] that explains behaviour of the Glu303Gln mutant. Retarded levan synthesis by Trp109Arg mutant was also expected as the equivalent mutant of *Z. mobilis* levansucrase (Trp80Arg) is also hampered in levan synthesis [6]. However, Trp109 replacement with Phe restored the ability for levan synthesis, achieving even increased production level of the fructan compared to wild-type Lsc3 (Figure 6). Levan production was very slow in case of mutants Trp109Ala, His113Ala, His113Gln and Glu236Gln. The Glu110Asp, Asp300Ala, Asp333Ala and Asp333Asn mutants were also somewhat hampered in levan synthesis (Figure 6).

Figure 5 shows that substitution of Asp333 in Lsc3 does not specifically reduce FOS production. Yet, levan synthesis is significantly decreased even in case of Asp333Asn mutant which behaves quite similarly to the wild-type protein regarding the V_max_ of sucrose-splitting reaction and FOS production (Figure 5 and Figure 6). Thus Asp333 can be specifically important for prolongation of the fructosyl chain. 

### 2.6. Thermostability Assay of Levansucrases Using Two Different Approaches

Enzymes applied in biotechnology should possess a high catalytic activity and long shelf-life. Bacterial extracellular proteins (including levansucrases) are suited for commercial applications as in nature they have to withstand harsh environmental conditions. We have shown earlier that Lsc3 protein has very high catalytic activity and long-term stability [21]. Previously we evaluated stability of the Lsc3 preparation during 30 days of storage at 37 °C in McIlvaine’s buffer (pH 6.0) with no loss of catalytic activity [21]. In our current study we further extended the storage time and report that during 140 days of incubation at 37 °C, Lsc3 retained 50% of initial sucrose-splitting activity. If the same preparation was kept at 50 °C, total catalytic activity dropped more rapidly—after 32 days the residual activity was only 6% of the initial activity. In current experiments, we kept the protein in 100 mM MES buffer (pH 6.5) supplemented with 150 mM NaCl. 

Thermofluor is a high-throughput method for protein characterization based on differential scanning fluorimetry [35]. In case of levansucrases, it has earlier been used for optimization of crystallization conditions of *Erwinia amylovora* levansucrase [8]. After having determined the optimal buffer conditions for wild-type Lsc3 we performed a Thermofluor assay on the panel of thirty seven levansucrase variants. The Thermofluor assay allows the determination of the melting temperature (T_m_) of the protein. Increase of temperature promotes unfolding of the protein and T_m_ is defined as the midpoint of the unfolding transition. A shift in T_m_ indicates a change in stability of the protein. The T_m_ for Lsc3 according to Thermofluor assay was 65.4 °C. The corresponding value of *E. amylovora* levansucrase was 57 °C and unfolding of this protein started at temperature above 45 °C [8]. In the assay, buffer conditions (100 mM HEPES buffer; pH 7.5 with 100 mM NaCl) were slightly different than those of our assay. When we conducted the Thermofluor assay of Lsc3 in 100 mM HEPES buffer (pH 7.5), the T_m_ value of it was only one degree lower than in 100 mM MES buffer (pH 6.5). Based on that we assume Lsc3 is substantially more thermostable than levansucrase of *E. amylovora*.

**Figure 7:**
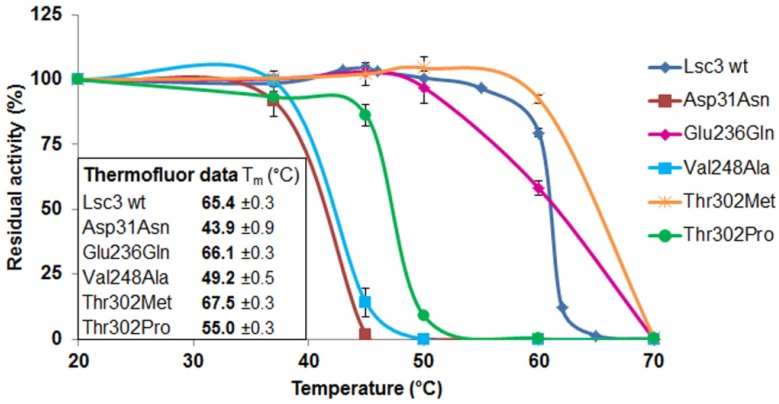
Thermal inactivation assay of purified wild-type Lsc3 and the mutant proteins Asp31Asn, Glu236Gln, Val248Ala, Thr302Met and Thr302Pro. After incubation of 5 μM of a levansucrase in McIlvaine’s buffer (pH 6.0) at a selected temperature, its residual activity was measured according to release of glucose from 100 mM sucrose [4]. The activity of a preparation kept at 20 °C was used as reference for 100% of activity. Inner panel shows T_m_ values obtained by the Thermofluor assay. The mean values of at least two parallel experiments and respective standard deviations are shown.

Most Lsc3 mutants had T_m_ values close to the wild-type protein, ranging from 62 to 66 °C. The mutants Asp31Asn, Val248Ala and Thr302Pro had much lower T_m_ than the wild-type Lsc3, whereas the mutant Thr302Met had enhanced stability—its T_m_ was 67.5 °C. We then performed a traditional thermal inactivation assay with wild-type Lsc3 and the mutants Asp31Asn, Glu236Gln, Val248Ala, Thr302Met and Thr302Pro. The proteins were incubated in McIllvaine’s buffer (pH 6.0) for 30 min at a temperature ranging from 20 to 70 °C, cooled on ice and residual total catalytic activity was determined by measuring release of glucose from 100 mM sucrose at 37 °C (see Section 3.10). As a result, mutants with reduced T_m_ values according to the Thermofluor assay also showed decreased thermostability in a traditional thermal inactivation study (Figure 7). We therefore conclude that Thermofluor can be used as a high-throughput tool to evaluate thermostability of levansucrases and their mutant variants.

### 2.7. Structure-Function Analysis of Novel Lsc3 Mutants

In the current work we introduce fourteen novel mutants of the Lsc3 protein of *P. syringae* pv. tomato DC3000. Phenotype of these mutants will be discussed according to the location of mutated residue, starting from N-terminus of the protein.

The Asp31Asn substitution originates from a random multiple mutant of Lsc3, MutG. *E. coli* expressing MutG from a plasmid had nonmucoid colonies on sucrose-containing agar medium. This mutant Lsc3 variant had four substitutions: Asp31Asn, Glu252Gly, Asp300Asn and Cys371Trp [21]. Based on MutG, two single mutants, Asp300Asn [21] and Asp31Asn were designed. Position corresponding to Asp31 is not strictly conserved—levansucrases of different bacteria and archaea have also His, Ala, Asn or Glu at this position (see Figure S2 in Supplementary materials). The Asp31Asn mutant has the lowest thermostability from mutants of the current work (Figure 7). It suggests that N-terminal domain of Lsc3 plays a role in the stability of the whole protein. The Asp31Asn substitution probably interferes with proper folding of the protein. According to crystal structure data of *G. diazotrophicus* and *B. subtilis* levansucrases, stable folding of levansucrases is provided through coordinated packing of N- and C-termini of the polypeptide. Similarly to *B. subtilis* levansucrase, the N-terminus of the *G. diazotrophicus* LsdA protein runs along the perimeter of blade IV forming a clamp-like loop that adds a fifth β-strand to blade III [5]. Notably, the amino acids of LsdA that comprise additional stabilizing β-strand to blade III are in close proximity of the “RADAL” motif [5] that harbours aspartate (underlined) corresponding to Asp31 of Lsc3.

The Trp109Arg mutant of Lsc3 is a counterpart of Trp109Arg mutant of *Z. mobilis* levansucrase, which displayed largely reduced levan synthesis [6]. We also made additional Trp109 substitutions with Ala and Phe. Figure 5 and Figure 6 show that the Trp109Arg and Trp109Ala mutants display a drastic reduction of levan and FOS synthesis. Some levansucrases, such as the G. *diazotrophicus* LsdA (see Figure 1), have a Phe at the corresponding position. This explains why substitution of Trp109 with Phe restored the wild-type phenotype to Lsc3. Replacement of Trp109 with Phe even slightly enhanced levan synthesis (Figure 6). We hypothesize that Phe in this position slightly changes the architecture of catalytic centre causing the increase in donor and acceptor binding. Therefore fructan chains are elongated more efficiently. There is no evidence based on crystal structures of levansucrases that Trp or Phe in the respective position directly interact with the substrate [1,5]. To make further conclusions, additional evidence from crystal structures and data on product spectra of levansucrases and their mutants are needed. According to Betts and Russell [36], change of Trp to Phe is favoured in proteins, whereas change to Ala or Arg is not. We assume that Trp109 participates in forming of +2 subsite of the substrate-binding pocket of Lsc3 (see Figure 2). As sucrose-splitting activities of Trp109Arg and Trp109Ala mutants are also strongly reduced (Figure 4 and Figure 5), Trp109 is probably also involved in binding of the substrate at +1 and/or −1 subsites or contributing to substrate cleavage reaction. This assumption is supported by the fact that the Trp109Arg mutant has strongly reduced affinity for sucrose—the K_m_ is increased from 18.5 to 249 mM (Table 1).

The Glu110Asp mutant was designed by us to study catalytic significance of a neighbouring position of Trp109. As shown in Figure 1, this position is not conserved in levansucrases. For example, respective residue in LsdA of *G. diazotrophicus* is Asp. Our data on the catalytic properties of Glu110Asp mutant shows no significant changes compared to the wild-type protein. Yet, velocity of levan synthesis was reduced and K_m_ for sucrose was increased about by 3 times (Figure 6, Table 1). Importantly, when Glu110 was changed to Ala, the protein completely precipitated and could not be purified by Ni^2+^-affinity chromatography (data not shown) suggesting the importance of the position.

The His113Gln substitution was detected by us in a random double mutant (MutE) of Lsc3. This mutant carried an additional substitution, Val195Ile, and *E. coli* expressing this protein had nonmucoid colonies on a sucrose-containing agar plate. We chose to design the His113Gln mutant due to its proximity to a nucleophile Asp62 on the 3D model and a high conservation score. Figure 2 shows that homologues of His113 residue in levansucrases reside close to fructose residue (binds at −1 subsite) and galactose residue (binds at +2 subsite) of the raffinose molecule bound to the enzyme. Crystal structure of *Arthrobacter* sp. K-1 fructosyltransferase in complex with fructose (PDB: 3VSS; [37]) also shows that His147 in *Arthrobacter* sp. K-1 enzyme (a homologue of His113 of Lsc3) is at hydrogen-bondage distance from bound fructose. Notably, the *Arthrobacter* sp. K-1 enzyme is structurally similar to levansucrases [23,37]. To verify the importance of His113 in the catalysis, we additionally constructed the His113Ala mutant. Like His113Gln substitution, it had a very strong effect on sucrose-splitting reaction and levan synthesis (Figure 4 and Figure 6). Notably, the Gln substitution had an even stronger negative effect on sucrose cleavage and FOS synthesis (Figure 5). We predict that His113 participates in the binding of both fructosyl donor (at −1 subsite) and acceptor (at +2 subsite) to Lsc3. Importantly, this is the first report of catalytic importance of this position in levansucrases.

The Glu146Gln variant of Lsc3 was constructed to mimic the Glu117Gln mutant (see Figure 1) of *Z. mobilis* levansucrase. The Glu117Gln mutant had decreased affinity for sucrose and increased transfructosylating activity [6]. According to our results, the Glu146Gln mutant of Lsc3 displayed a two-fold increase in K_m_ towards sucrose, whereas significantly enhanced transfructosylation was not detected (Table 1; Figure 6). Still, our assay showed that the Glu146Gln mutant produced more FOS per mg of protein than the wild-type Lsc3 (Figure 5), whereas levan production was reduced (Figure 6). Typically, levansucrases have a Glu at position corresponding to Glu146 of Lsc3, but the *Arthrobacter* sp. K-1 β-fructofuranosidase which has a high transfrucosylating activity, has a Gln [37]. We conclude that substitution of Glu146 in Lsc3 with Gln favours synthesis of FOS at the expense of levan.

The Glu236Gln mutant of Lsc3 corresponds to Glu211Gln mutant of *Z. mobilis* levansucrase which retained only 28% of hydrolyzing activity and 17% of transfructosylating activity of the wild-type protein [6]. The Glu236Gln mutant of Lsc3 showed decreased transfructosylation and ability to bind sucrose—the K_m_ was increased 14.4-fold (see Table 1 and Figure 5 and Figure 6). The position corresponding to Glu236 in Lsc3 is completely conserved among levansucrases (see Figure 1 and Figure 2). In-depth analysis of crystal structures of *B. subtilis* levansucrase SacB complexed with substrates (PDB IDs: 1PT2 in complex with sucrose and 3BYN with raffinose [10,38]) revealed that Glu262 locates close to Arg246 of the RDP motif that participates in binding of the substrate at both the −1 and +1 subsites. According to both crystal structures, Glu262 forms hydrogen bonds with Arg246 and also with fructose and glucose residues of the substrate over a water molecule. We assume that Glu236 contributes to stabilization of the substrate in the active site.

The Val248Ala mutant of Lsc3 is a counterpart of Val223Ala mutant of *Z. mobilis* levansucrase that displayed a tenfold decrease of the k_cat_ whereas the TA value remained the same [6]. The V_max_ of sucrose-splitting reaction of Val248Ala mutant of Lsc3 was 39% of the wild-type level when measured at 37 °C (Figure 5). However, the T_m_ value of the Val248Ala mutant was 16 degrees lower than that of the wild-type enzyme and its reduced thermostability became also evident in a traditional thermal inactivation assay (Figure 7). We assume that Val248Ala substitution affects the folding of Lsc3 protein and thereby its stability and catalytic activity.

We have previously described the Asp300Asn mutant originating from random mutagenesis [21]. This substitution slightly reduces the affinity of the protein for sucrose. An interesting feature of the Asp300Asn mutant is the production of long-chain FOS, with DP up to 10, whereas the wild-type Lsc3 synthesizes FOS with DP up to 7 [21,23]. The Asp300Ala mutant addressed in the current work behaved similarly to wild-type Lsc3, producing FOS with DP up to 8 (Table 1). Thus, for a yet unknown reason, the presence of Asn at position 300 of Lsc3 promotes synthesis of long-chain FOS.

The Asp333Asn mutant of Lsc3 was constructed to mimic the Asp308Asn mutant of *Z. mobilis* levansucrase. The Asp308Asn mutant had slightly decreased transfructosylation ability and about four-fold decreased affinity for sucrose [6]. This position is highly conserved in levansucrases (Figure 1). Yet, in *Arthrobacter* sp. K1 enzyme, a Glu, and in *B. subtilis* SacB an Asn (Asn372) is found at the same position (Figure 1). Aside of making the Asp333Asn mutant, we also constructed respective Ala substitution variant. V_max_ of sucrose-splitting reaction of the Asp333Ala mutant was four-fold lower compared to wild-type enzyme and levan synthesis was also significantly reduced. The Asp333Asn mutant behaved much more similarly to wild-type Lsc3 than the Asp333Ala (Figure 5 and Figure 6). We suppose that Asp333 can be involved in substrate binding and elongation of the fructan chain.

## 3. Experimental

### 3.1. Construction of Levansucrase Mutants

Mutant variants of levansucrase *lsc3* gene were constructed using primer-based site-specific mutation strategy as reported earlier [21,23]. Mutagenic oligonucleotides and respective amino acid replacements are listed in Table S1 of Supplementary materials. Cloning of the genes into the expression vector pURI3 [39] was performed as shown in [21,23]. Plasmid DNA was purified using FavorPrep^TM^ Plasmid Extraction Mini Kit (Favorgen Biotech Corp., Ping-Tung, Taiwan) and the mutations were verified by DNA sequencing.

### 3.2. Cultivation of Bacteria and Purification of Recombinant Levansucrases

Transformed *E. coli* was grown in LB broth containing ampicillin (Amp) 0.15 mg/mL at 37 °C. Mutated and wild-type Lsc3 proteins were overexpressed in *E. coli* BL21(DE3) [40]. Purification of N-terminally His-tagged proteins was performed as in [21]. For phenotypic evaluation of *E. coli* BL21(DE3) transformants expressing levansucrase variants, they were grown overnight at 37 °C on MacConkey + Amp medium containing 10% sucrose and 1 mM isopropyl *β*-D-1-thiogalactopyranoside (IPTG). The plates were further kept at room temperature (23 °C) until levan synthesis (slime production) and acid production (pink colour) from the sugar became evident.

### 3.3. Permeabilization of E. coli Cells and Sucrose-Splitting Activity Assay of Levansucrases on Permeabilized Bacteria

The levansucrase-expressing transformants of *E. coli* BL21(DE3) were grown overnight in LB-Amp broth on a sterile 96-well flat-bottom transparent polystyrol microplate (CELLSTAR, Greiner Bio-One, Frickenhausen, Germany) at 37 °C on a shaker (900 rpm) with 200 µL of the culture per well. Overnight cultures were diluted in LB-Amp broth to OD_600 nm_ of ~0.016 (respective value measured in a 1 cm-pathway cuvette is 0.05) and were further agitated on a shaker at 37 °C for 2 h. To induce levansucrase expression, IPTG was added to the wells (final concentration 0.5 mM) and the microplate was further incubated on a shaker at 22 °C for 20 h. Then, OD_600 nm_ values of the cultures were measured and they were diluted approximately 10 times in McIllvaine’s buffer (pH 6.0) to obtain the OD_600 nm_ value of approximately ~0.067 (respective value measured in a 1 cm-pathway cuvette is 0.2). Undiluted culture was used in case of clones with severely reduced sucrose-splitting activity.

Next, the cells were permeabilized with 0.1% cetyltrimethylammonium bromide (CTAB). Briefly, 50 μL of microplate-grown and appropriately diluted *E. coli* BL21(DE3) cultures were combined with 50 μL of 0.2% CTAB solution in McIllvaine’s buffer (pH 6.0) in wells of a new microplate and agitated (900 rpm) for 10 min at room temperature to permeabilize cellular membranes. Levansucrase reaction was then started by adding 50 μL of 300 mM sucrose (final concentration 100 mM) in McIllvaine’s buffer (pH 6.0). The reaction was conducted at 37 °C for 5 min and then stopped by transferring 10 μL of the reaction mixture into a new well containing 30 μL of Tris buffer (200 mM, pH 8.3), subsequently heated at 96 °C for 5 min and cooled on ice. Released glucose was visualized by adding 160 μL of Glucose liquicolor reactive (Human GmbH, Wiesbaden, Germany) and incubating the plate for 5 min at 37 °C. Absorbance of the reddish complex was measured at 500 nm. All absorbance values were recorded using Infinite M200 PRO microplate reader (Tecan Group Ltd., Männedorf, Switzerland) equipped with Tecan i-control 1.7 software. *E. coli* culture BL21(DE3) carrying the empty pURI3 plasmid served as a negative control for the assay. The sucrose-splitting activity was expressed as percentage of the respective normalized activity of the wild-type Lsc3. To normalize the activities, OD_600 nm_ values of cell suspensions in the wells were measured. Correction coefficients for mutants were calculated by dividing the OD_600 nm_ value of the wild-type to the OD value of the respective mutant.

### 3.4. Sucrose-Splitting Activity Assay on Microplates Using Purified Levansucrases

To measure the sucrose-splitting activity of purified levansucrases, proteins were diluted in McIllvaine’s buffer to contain 2 U/mL of the enzyme. Unit values were calculated from sucrose-splitting activity measurements with 100 mM sucrose at 37 °C [4,21]. Assay was conducted as in case of using permeabilized cells (see Section 3.3), but instead of cells, appropriately diluted purified proteins were used. 50 µL of purified protein preparation (final concentration in reaction mixture 0.67 U/mL; approximately 1.7 µg/mL in case of wild-type Lsc3) were combined with equal amount of McIllvaine’s buffer (pH 6.0). In case of mutants with extremely low sucrose-splitting activities, 10 µg of protein per well (66.7 µg/mL) was used. The reaction was started by adding 50 μL of 300 mM sucrose (final concentration 100 mM) in McIllvaine’s buffer and conducted for 5 min at 37 °C. Stopping of the reaction and measurement of glucose content were performed as described above (see Section 3.3). Glucose released from sucrose was quantified from respective calibration curve. Activity unit expresses the amount of glucose in µmoles produced per min per mg of levansucrase protein (U/mg). Activity value obtained with wild-type Lsc3 was taken as 100%.

### 3.5. FOS-production Assay of Levansucrases Using Permeabilized E. coli

Fifty µL of the levansucrase-expressing *E. coli* BL21(DE3) cultures were permeabilized as shown in Section 3.3. Then, 100 µL of 2 M sucrose (final concentration in a well 1 M) in McIllvaine’s buffer were added, the plate was sealed firmly to avoid evaporation and incubated without agitation at 37 °C for 20 h. The reaction was stopped by heating the microplate for 5 min at 96 °C. For HPLC analysis of polymerization products, the reaction samples were transferred from the microplate to microtubes and centrifuged for 10 min at 16,000 g. The supernatants were withdrawn and further heated for 10 min at 96 °C to completely inactivate the levansucrase protein. Mono- and oligosaccharides in the samples were analysed using HPLC (Section 3.9). *E. coli* culture BL21(DE3) carrying the empty pURI3 plasmid served as negative control for the assay.

### 3.6. FOS-production Assay of Levansucrases Using Purified Proteins

The FOS-production assay was conducted as described in [23]. Purified proteins (2.7 U/mL) were incubated in McIllvaine’s buffer (pH 6.0) containing 1.2 M sucrose at 37 °C for 20 h. The reactions were conducted in Eppendorf tubes, the final volume of the reaction mixture was 1 mL. In case of mutants with severely reduced catalytic activity (Trp61Ala, Trp61Asn, Asp62Ala, Trp109Arg, Asp219Ala, Arg304Cys, Glu303Ala, Glu303Gln), 100 µg/mL of purified protein was used in the reaction. The reaction was stopped by heating samples at 96 °C for 5 min. Mono- and oligosaccharides in the samples were analysed using HPLC (Section 3.9).

### 3.7. Levan Synthesis Assay of Levansucrases on Microplates Using Purified Proteins

Kinetics of levan synthesis from sucrose by the purified Lsc3 variants was recorded on microplates using 10 μg/mL (the final concentration in reaction mixture) of purified protein. The proteins were diluted in McIlvaine’s buffer (pH 6.0) and incubated at 37 °C on transparent flat-bottom polystyrole microplates in a total volume of 200 μL per well. The reaction was started by adding 2 M sucrose in McIlvaine’s buffer at final concentration 0.6 M. Microplates were incubated in Infinite M200 PRO microplate reader (Tecan Group Ltd.) at 37 °C and the turbidity (OD_400 nm_) values were recorded every 15 min during 12 h.

### 3.8. Determination of V_max_, K_m_ for Sucrose and Transfructosylating Activity of Purified Proteins

K_m_ and V_max_ of levansucrases were determined as reported previously [23]. For that, the initial velocities of sucrose-splitting reaction were determined at varied concentrations (from 5 to 1200 mM) of sucrose. Data was analysed using Enzyme Kinetics Module 1.1 of Sigma Plot 2001 (SYSTAT, San Jose, CA, USA). Protein concentration was determined by measuring the absorbance at 280 nm. The extinction coefficients were computed at ExPASy Proteomics Server.

Transfructosylating activity (TA) was calculated as percentage of fructose residues from reacted sucrose molecules incorporated into polymerization products [6,21,23].

### 3.9. Quantification of Sugars by HPLC Analysis

HPLC method was used to quantify glucose, fructose, sucrose and FOS content in the reaction mixtures [23,41]. Chromatography was performed on an Alltech Prevail Carbohydrate ES column (Grace, Deerfield, IL, USA) using Acquity UPLC system (Waters, Milford, MA, USA) coupled with evaporative light scattering (ELS) detector similarly as in [23]. The mobile phase consisted of LC-grade water (A) and acetonitrile (B). The gradient of solvent B was following: 70%–50% 15 min; 50% 10 min; 50%–70% 2 min; 70% 13 min at flow rate of 0.6 mL/min. d-glucose (Oriola, Espoo, Finland), d-fructose (Sigma-Aldrich, St. Louis, MO, USA), sucrose (Serva, Heidelberg, Germany), raffinose (Naxo, Tartu, Estonia), 1-kestose (Sigma-Aldrich) and nystose (Sigma-Aldrich) were used as standards. Calibration curves were made using standard solutions with concentrations ranging from 0.1 to 5 mg/mL. FOS in the range of degree of polymerization of 3–10 were calibrated against raffinose.

### 3.10. Thermofluor Assay of Thermostability of Levansucrases and Levansucrase Activity Assay of Thermostability

Thermal shift assay, also known as Thremofluor^®^ [35], was performed using a LightCycler^®^ 480 System (Roche, Basel, Switzerland). Combined channels (465–580 nm) were selected as suitable for the measurements using SYPRO^®^ Orange (Sigma-Aldrich) as a fluorescent dye. The samples (final amount 20 μL) contained 2 μM of the protein and 5× SYPRO^®^ Orange in 100 mM MES buffer (pH 6.5) with 150 mM NaCl. Samples were pipetted onto a Roche white LightCycler^®^ 480 multiwell plate for 96 samples and sealed with the foil as recommended by the manufacturer. The plate was centrifuged at room temperature at 200 g for 30 s to remove the air bubbles.

Temperature gradient from 27 to 95 °C was applied and data was recorded using LightCycler^®^ 480 software (release 1.5.0 SP3). The melting temperature (T_m_), used here as a thermostability characteristic of levansucrases, is defined as temperature at which half of the protein in the studied sample is unfolded. The T_m_ was specified as temperature at which the derivative curve apex had a minimum value. Mutants that differed significantly from the wild-type Lsc3 according to T_m_ were selected for traditional thermostability assay to obtain data for comparison. Purified Lsc3 mutants Asp31Asn, Glu236Gln, Val248Ala, Thr302Met, Thr302Pro and the wild-type Lsc3 (5 μM of protein in McIlvaine’s buffer; pH 6.0) were incubated at varied temperature (from 20 to 70 °C) for 30 min and then cooled on ice. Residual total levansucrase activity of the samples was measured at 37 °C by quantifying the release of glucose from 100 mM sucrose as in [4,21].

## 4. Conclusions

We conclude that cost-saving high-throughput methods are feasible for preliminary characterization of levansucrases and their mutant variants. Importantly, some features of levansucrases such as the sucrose-splitting activity and the ability to produce fructooligosaccharides, can be reliably evaluated on surfactant-treated recombinant *Escherichia coli* cells expressing the protein. Thermofluor method was used here for the first time to characterize thermostability of levansucrase mutants. Our current study provides new tools for the isolation, selection and characterization of levansucrases. These proteins are important as they can be used to synthesize β-2,6-linked fructooligosaccharides and levan, biological effects of which are yet poorly assayed compared with respective β-2,1-linked counterparts.

## Supplementary Materials

Supplementary materials can be accessed at: http://www.mdpi.com/1420-3049/19/6/8434/s1.
