# Supplementary material for: High-Throughput Assay of Levansucrase Variants in Search of Feasible Catalysts for the Synthesis of Fructooligosaccharides and Levan
Source: Molecules. 2014 Jun 20;19(6):8434–55. doi: 10.3390/molecules19068434 (PMC6271050; doi:10.3390/molecules19068434)
Supplement: Supplementary file 1 [file molecules-19-08434-s001.pdf]

Supplementary Materials

Table S1. List of Lsc3 mutants and mutagenic oligonucleotides used in the study. Mutated codon is indicated on grey background and mutated nucleotides are underlined. Publications addressing the mutants are indicated. Some of mutants were designed to mimic respective mutants of *Zymomonas mobilis* levansucrase [1]. Mutagenesis was performed as shown in section 3.1 of the main text and in Ref [2].

Mutation in Lsc3	Mutagenic Primer	Primer Sequence (5'→3')	Origin
Asp31Asn (D31N)	D31NRev	ACCTTCAGCGCATTGGCG	This work
Trp61Ala (W61A)	W61ARev	ATGGTGTCCGCGATGAATACCG	[3]
Trp61Asn (W61N)	W61NRev	ATGGTGTCGTTGATGAATACCG	[3]
Asp62Ala (D62A)	D62ARev	CATGGTGGCCCAGATGAAT	[3]
Thr63Ala (T63A)	T63ARev	AGCGGCATGGCGTCCCAGATG	[3]
Leu66Ala (L66A)	L66ARev	TCTCGCGCCGGCATGGTG	[3]
Trp109Ala (W109A)	W109ARev	TGTCGATCTTCCGCGTCACGC	This work
Trp109Phe (W109F)	W109FRev	TGTCGATCTTCAAAGTCACGC	This work
Trp109Arg (W109R)	W109RRev	TGTCGATCTTCCCGGTCACGC	[1]; this work
Glu110Asp (E110D)	E110DRev	ATGTCGATCGTCCCAGTCACGC	This work
His113Ala (H113A)	H113ARev	CGCGGCCAGCTCGATCTTCC	This work
His113Gln (H113Q)	H113QRev	GCGTGCGCGGCCTTGTCG	This work
Glu146Gln (E146Q)	E146QRev	GGTGTGCCCGCCCACTGTCGAGTGGTGG	[1]; this work
Asp219Ala (D219A)	D219ARev	ACTGGGGGCGCGAAAGTTC	[3]
Pro220Ala (P220A)	P220ARev	ATGAACGGACTGGCGTCGCG	[3]
Asp225Ala (D225A)	D225ARev	CATCATTAGGGGCAATGAACGGAC	[3]
Asp225Asn (D225N)	D225NRev	CATCATTAGGATTAATGAACGGAC	[3]
Glu236Gln (E236Q)	E236QRev	TCACCGGCGACATTGCCCTGAAACACCA	[1]; this work
Val248Ala (V248A)	V248AFw	TTCGCACACAGCAGGGGTTGC	[1]; this work
Asp300Ala (D300A)	D300AFw	GGCGTGAATGCGCAGACCGAG	This work
Asp300Asn (D300N)	D300NFw	TGAATAATCAGACCGAGCGCC	[2]
Gln301Ala (Q301A)	Q301AFw	TGAATGATGCGACCGAGCGC	[3]
Gln301Glu (Q301E)	Q301EFw	TGAATGATGAGACCGAGCGC	[3]
Thr302Met (T302M)	T302MFw	AATGATCAGATGGAGCGCCC	[3]
Thr302Pro (T302P)	T302PFw	AATGATCAGCCCGAGCGCCCG	[2]
Glu303Ala (E303A)	E303AFw	TGATCAGACCGCGCGCC	[3]
Glu303Gln (E303Q)	E303QFw	TGATCAGACCCAGCGCCCGC	This work
Arg304Ala (R304A)	R304AFw	TCAGACCGAGGCCCCGCACTATG	[3]
Arg304Cys (R304C)	R304CFw	TCAGACCGAGTGCCCGCACTATG	[3]
His306Ala (H306A)	H306AFw	AGCGCCCGGCCTATGTTTTTCAAG	[3]
His321Leu (H321L)	H321LFw	CACCATCAGTCTGAAGTTCACGTATGCC	[2]
His321Lys (H321K)	H321KFw	CACCATCAGTAAAAAGTTCACGTATGCC	[2]
His321Arg (H321R)	H321RFw	CACCATCAGTCGCAAGTTCACGTATGCC	[2]
His321Ser (H321S)	H321SFw	CACCATCAGTAGCAAGTTCACGTATGCC	[2]
Asp333Ala (D333A)	D333AFw	GGGCCAGCGGGGGTGTACGG	This work
Asp333Asn (D333N)	D333NFw	GGGCCAAACGGGGTGTACGG	[1]; this work

Figure S1. Typical high-performance liquid chromatography (HPLC) analysis of reaction products of Lsc3 wild-type (Lsc3 wt; upper panel) and a mutant with reduced catalytic activity (e.g. Leu66Ala; lower panel) conducted using CTAB-permeabilized cells (violet line) or purified protein (red line). Peaks corresponding to fructose (F), glucose (G), sucrose (S) and FOS with degree of polymerization (DP) 3 to 6 are designated. Detection sensitivity was reduced to fully accommodate the peaks of glucose and sucrose. Due to that, FOS with DP > 5 cannot be seen on the chromatogram. Lsc3 wt and Leu66Ala mutant both synthesize FOS with DP up to 7 (see Table 1 of the main text and Ref [3]). Reactions were performed as shown in sections 3.5 and 3.6 of the main text and products were analyzed by HPLC as in section 3.9 and Ref [3]. LSU – light scattering unit determined on evaporative light scattering (ELS) detector.

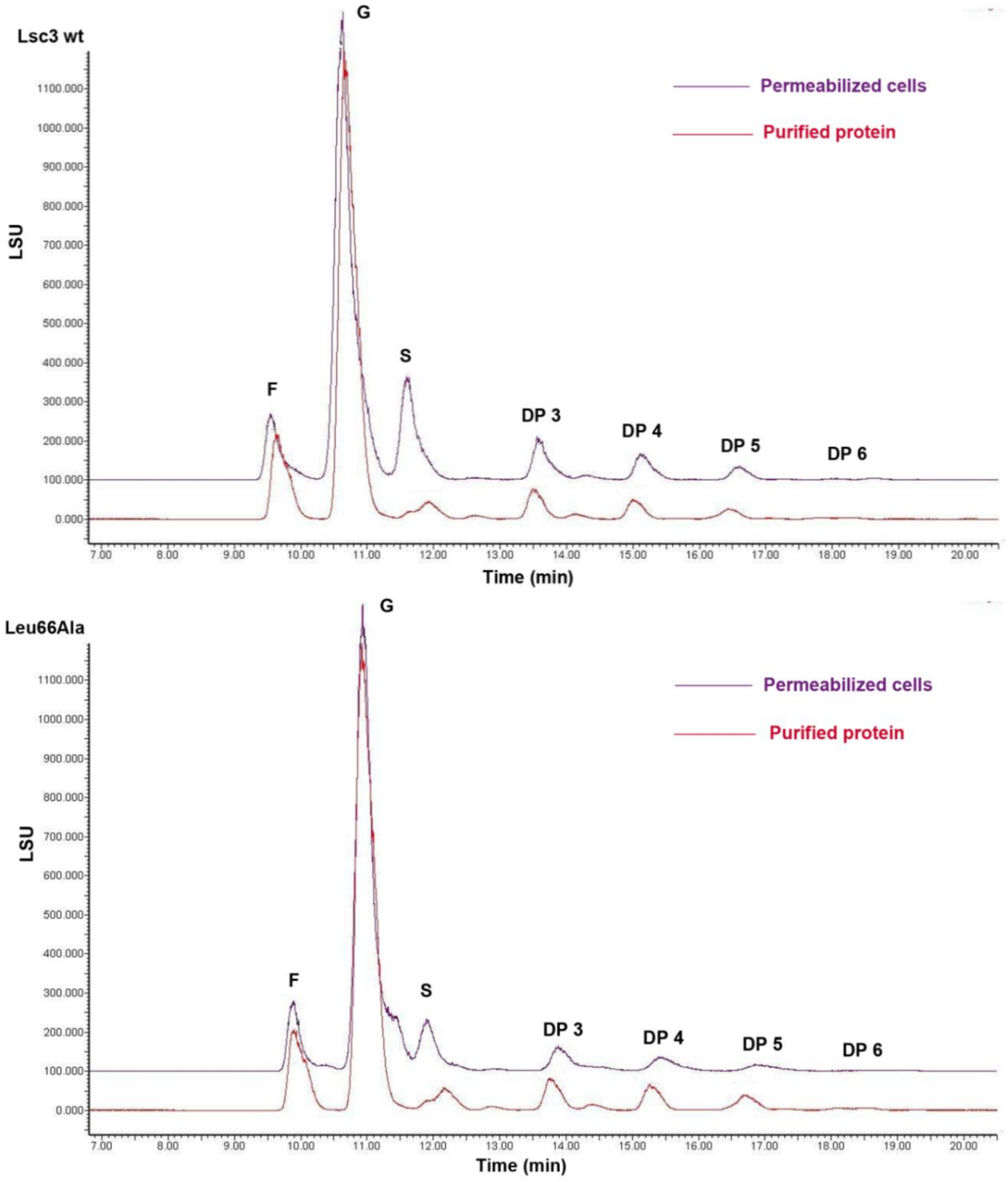

Figure S2. Alignment of protein sequences of 22 levansucrases. Fragment containing the position corresponding to Asp31of *Pseudomonas syringae* pv. tomato levansucrase Lsc3 is shown and Asp31 residue is indicated as # above the MUSCLE [4] alignment. Sequences were extracted from UniProt [5] database. Levansucrase protein sequences of following bacteria are shown: *Pseudomonas syringae* pv. phaseolicola, *Serratia odorifera*, *Pseudomonas fluorescens*, *Rahnella aquatilis*, *Erwinia amylovora*, *Pseudomonas chlororaphis* subsp. *aurantiaca*, *Zymomonas mobilis*, *Gluconobacter oxydans*, *Gluconacetobacter diazotrophicus*, *Beijerinckia indica*, *Burkholderia phymatum*, *Burkholderia pseudomallei*, *Clostridium acetobutylicum*, *Streptomyces viridochromogenes*, *Cellulomonas flavigena*, *Actinomyces viscosus*, *Halorubrum lacusprofundi*, *Halalkalicoccus jeotgali*, *Halomicrobium mukohataei*, *Haloarcula marismortui*, *Bacillus subtilis*.

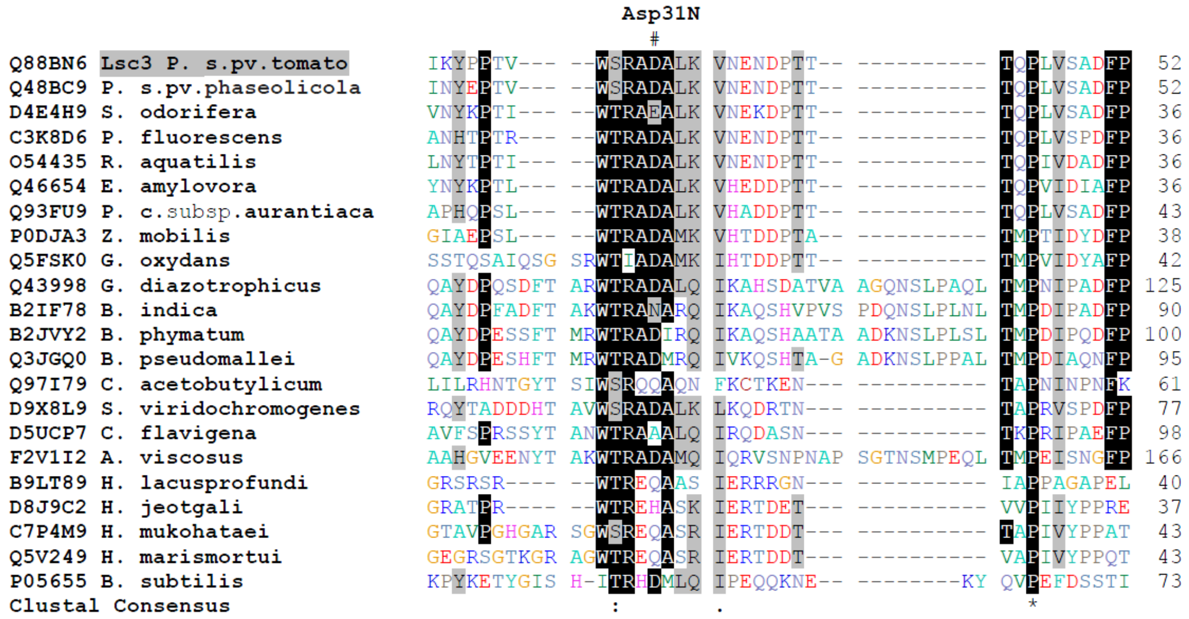

References

1. Yanase, H.; Maeda, M.; Hagiwara, E.; Yagi, H.; Taniguchi, K.; Okamato, K. Identification of functionally important amino acid residues in *Zymomonas mobilis* levansucrase. *J. Biochem.* **2002**, *132*, 565–572.
2. Visnapuu, T.; Mardo, K.; Mosoarca, C.; Zamfir, A.D.; Vigants, A.; Alamäe T. Levansucrases from *Pseudomonas syringae* pv. tomato and *P. chlororaphis* subsp. *aurantiaca*: Substrate specificity, polymerizing properties and usage of different acceptors for fructosylation. *J. Biotechnol.* **2011**, *155*, 338–349.
3. Mardo, K.; Visnapuu, T.; Vija, H.; Elmi, T.; Alamäe, T. Mutational analysis of conserved regions harboring catalytic triad residues of the levansucrase protein encoded by the *lsc-3* gene (*lsc3*) of *Pseudomonas syringae* pv. tomato DC3000. *Biotechnol. Appl. Biochem.* **2014**, *61*, 11–22.
4. Edgar, R.C. MUSCLE: multiple sequence alignment with high accuracy and high throughput. *Nucleic Acids Res.* **2004**, *32*, 1792–1797.
5. The UniProt Consortium. Activities at the universal protein resource (UniProt). *Nucleic Acids Res.* **2014**, *42*, 191–198.
